# Correlates of reported rotavirus vaccination among young Indonesian children: a cross-sectional analysis of the Indonesia Family Life Survey (IFLS-5)

**DOI:** 10.3389/fpubh.2026.1839949

**Published:** 2026-09-18

**Authors:** Rano K. Sinuraya, Yasmin Fatinah, Auliya A. Suwantika, Irma M. Puspitasari

**Affiliations:** 1 Department of Pharmacology and Clinical Pharmacy, Faculty of Pharmacy, Universitas Padjadjaran, Sumedang, West Java, Indonesia; 2 Center of Excellence for Pharmaceutical Care Innovation, Universitas Padjadjaran, Sumedang, West Java, Indonesia

**Keywords:** child health, immunization, Indonesia, Indonesia Family Life Survey, out-of-pocket payment, rotavirus vaccine, vaccination coverage, vaccine uptake

## Abstract

**Background:**

Rotavirus is a major cause of childhood diarrheal disease, but evidence on vaccine uptake in Indonesia is limited. During the study period the vaccine was available only privately, on a self-paid basis. This study examined factors associated with uptake before national introduction in 2023, characterizing who is reached when a vaccine is available but not financed.

**Methods:**

We analyzed the fifth wave of the Indonesia Family Life Survey (IFLS-5, 2014–2015), including children aged 1–5 years with caregiver-reported rotavirus vaccination status. Hierarchical binary logistic regression was used. Child age, equivalent to birth cohort in this single-wave survey and thus indexing each child’s eligibility window, was included in every model. Complete-case analysis was primary; its identifying assumption was tested directly, and four sensitivity analyses were added: inverse-probability weighting, a tipping-point analysis, multiple imputation (m = 20), and Firth penalized regression restoring two variables affected by quasi-complete separation.

**Results:**

Among 3,320 children, 287 (8.64%) had received at least one dose. In the fully adjusted complete-case model, higher odds were observed for complete basic immunization (AOR 2.71, 95% CI 1.73–4.24) and postnatal care (AOR 1.57, 95% CI 1.17–2.11), and lower odds for children in Islam-headed households (AOR 0.31, 95% CI 0.21–0.47) and in Bali and Nusa Tenggara (AOR 0.51, 95% CI 0.31–0.84), with higher uptake in Sulawesi than Java (AOR 2.81, 95% CI 1.64–4.81). These four findings were unchanged under multiple imputation and Firth penalization. Associations with male sex, secondary maternal education, maternal age above 30 years and paternal age 41–50 years did not persist under imputation and are not robust. Child age, equivalently birth cohort, was not associated with uptake (AOR 1.02, 95% CI 0.91–1.13), and the religion association differed significantly across regions (*p* = 0.002).

**Conclusion:**

Uptake was low and robustly associated with prior use of preventive child health services and with sociocultural and geographic context. Because the religion association varied markedly across regions, it is interpreted as a marker of local context rather than a causal effect of religious belief. Several weaker associations were not robust to multiple imputation. Findings are associations, not causal relationships.

## Introduction

Vaccination is widely recognized as one of the most effective public health interventions for preventing infectious diseases and reducing child morbidity and mortality (1). Despite its proven benefits, achieving high vaccine coverage remains a challenge in many low- and middle-income countries, where barriers related to access, affordability, and public acceptance may reduce the impact of immunization programs (2). For countries with large and diverse populations such as Indonesia, understanding the factors that influence vaccine uptake is essential for improving child health outcomes and strengthening immunization strategies (3).

Diarrhea remains an important cause of illness and death among children in Indonesia, and rotavirus is a major contributor to severe acute gastroenteritis in early childhood (4). The national health profile has identified diarrhea as one of the leading causes of death in the post-neonatal period, underscoring the need for effective preventive measures. In this context, rotavirus vaccination has considerable public health value because it can reduce severe disease, hospitalizations, and the broader burden of childhood diarrhea (5). Previous studies in Indonesia have also highlighted the importance of rotavirus prevention and called for wider implementation of rotavirus vaccination in the national program (4, 5).

However, the uptake of a vaccine depends on more than its clinical effectiveness alone. In this study, we distinguish between several related but distinct concepts. Vaccine *uptake* refers to whether a child actually received the vaccine; vaccine *access* refers to the physical and structural availability of vaccination services; vaccine *affordability* refers to the household’s ability to bear the cost of a self-paid vaccine; and vaccine *acceptance* refers to parental willingness to vaccinate once access and affordability are in place. These dimensions overlap but are not identical, and the present analysis focuses on uptake rather than on acceptance or hesitancy per se, because IFLS-5 did not collect direct measures of parental knowledge, provider recommendation, willingness to pay, or vaccine availability. A growing body of literature has shown that vaccine acceptance and hesitancy are influenced by demographic characteristics, education, accessibility, cost, perceptions of risk, trust in health authorities, and concerns about vaccine safety and effectiveness (6, 7).

These issues may be especially important for newer vaccines. During the IFLS-5 period (2014–2015) (8), rotavirus vaccine was not part of Indonesia’s national immunization program and was available only through the private sector on a self-paid basis. This had direct implications for access, cost, eligibility, and provider recommendation: families had to be aware of the vaccine, able to reach a private provider, willing to pay out of pocket, and advised to vaccinate, all of which could limit uptake even where willingness was present. Rotavirus vaccine was subsequently introduced into the national immunization program in 2023, beginning in South Sulawesi and targeting infants from 2 months of age (8, 9). The IFLS-5 data therefore capture a pre-introduction baseline that remains valuable for understanding the determinants of early, self-paid uptake and for informing the scale-up of the now-national program.

The value of these data, however, extends beyond rotavirus and beyond the pre-2023 period. Because the vaccine was then available but unsubsidized, the situation studied here is a general one: uptake of a vaccine that families must learn about, physically reach, and pay for themselves, with no programmatic prompt and no financial protection. Several vaccines in Indonesia remain in precisely this position today. Evidence on which children are reached, and which are missed, when availability exists without financing is therefore directly relevant to current immunization policy, and not only to the interpretation of the rotavirus program now under way. We accordingly treat this analysis as serving two purposes: establishing a pre-introduction baseline for rotavirus specifically, and characterizing the correlates of uptake under availability without financing more generally.

Evidence from different settings shows that vaccine decisions are shaped by socioeconomic and family-level characteristics, and disparities in socioeconomic status have been linked to unequal vaccination uptake in low- and middle-income countries (10, 11). In Indonesia, parental knowledge and attitudes toward childhood vaccination are generally positive, but evidence on rotavirus vaccine uptake specifically remains limited (3), leaving it unclear which children are more likely to be vaccinated and how social, cultural, and geographic factors shape parental decisions. Understanding these determinants is particularly important given considerable regional variation in healthcare access and sociocultural context across Indonesia. The IFLS offers a population-based dataset covering approximately 83% of the Indonesian population, providing an empirical baseline before national introduction against which post-introduction progress can be measured. This study therefore aimed to identify factors associated with rotavirus vaccine uptake among young children in Indonesia using data from the fifth wave of IFLS.

## Methods

### Rotavirus immunization in Indonesia

Rotavirus vaccine is an oral vaccine administered in early infancy. Internationally, licensed rotavirus vaccines are given as a two- or three-dose series beginning at approximately 6 weeks of age, with the schedule completed within the first months of life. In Indonesia, rotavirus vaccine was available only privately during the IFLS-5 period and had to be purchased out of pocket. Rotavirus vaccine was introduced into the national immunization program in August 2023, initially in South Sulawesi and subsequently expanded nationally, following recommendations from the World Health Organization and the Indonesian Technical Advisory Group on Immunization (ITAGI). Under the national program, the vaccine is given as oral drops in a three-dose schedule starting at 2 months of age, with doses spaced about 4 weeks apart and the final dose given by approximately 6–7 months of age (8, 9, 12). Because rotavirus vaccination is biologically and programmatically an infant vaccine, the present analysis was restricted to young children, as described below.

### Study design and data source

This was a cross-sectional analysis of secondary data from the fifth wave of the Indonesia Family Life Survey (IFLS-5), conducted in 2014–2015. IFLS is a large longitudinal household survey in Indonesia that collects detailed information on demographic, socioeconomic, behavioral, and health-related characteristics of individuals and families. The survey covers households in 13 provinces representing about 83% of the Indonesian population, and the design and implementation of IFLS-5 have been described in detail elsewhere (8, 13). Parent- and child-level data were extracted from the IFLS-5 database and merged for analysis.

### Study population

The IFLS-5 childhood vaccination module records general immunization information for children across a wider age range (up to 14 years); however, information on rotavirus vaccination was available only for children aged 1–5 years at the time of the survey. These children therefore constituted the analytic sample (*n* = 3,320). Because rotavirus vaccine is given in early infancy, this group is well suited to the analysis: the eligibility window had occurred relatively recently, which reduces caregiver recall error, and because the sample spans only a narrow early-childhood band it avoids the birth-cohort heterogeneity that a wider age range would introduce. Rotavirus vaccination status was determined based on parent or caregiver report. Only children with available information on rotavirus vaccination status were included in the analytic sample.

Because eligibility for rotavirus vaccine is determined by date of birth rather than by age at interview, each child’s birth cohort was also considered explicitly. IFLS-5 fieldwork was carried out in late 2014 and during 2015, so children aged 1–5 completed years at interview belonged to birth cohorts spanning approximately 2009 to 2014, and their eligibility windows, from about 6 weeks to 6 or 7 months of age, fell between roughly 2009 and 2015. Every child in the analytic sample was therefore born, and passed through the age of eligibility, well before rotavirus vaccine entered the national immunization program in 2023, and all faced the same policy regime of exclusively private, self-paid provision. Birth cohort in these data consequently does not proxy differential programmatic eligibility, and no child was age-ineligible for the vaccine during the period in which it was obtainable. Birth cohort may nonetheless capture secular change over 2009–2015 in private-market availability, provider familiarity with the product, and public awareness of the vaccine, as well as differences in the interval between the eligibility window and the interview, which is relevant to the accuracy of caregiver recall. For these reasons child age at interview (equivalently, birth cohort) was carried as a covariate in every model, as described below.

### Outcome variable

The outcome variable was rotavirus vaccine uptake, defined as receipt of at least one dose of rotavirus vaccine, and was coded as a binary variable (yes/no). In IFLS-5, this information was based on parent or caregiver report; the survey did not record the number of doses received or provide vaccination-card verification for rotavirus vaccine. The outcome therefore represents caregiver-reported receipt of at least one dose (partial uptake) rather than completion of the rotavirus vaccine schedule. Only children with available information on rotavirus vaccination status were included in the analysis.

### Independent variables

Independent variables were selected based on previous literature, conceptual relevance, and availability in IFLS-5. Twenty-eight candidate variables were initially identified and grouped into five domains: child-related factors, maternal/perinatal healthcare factors, maternal sociodemographic factors, household/contextual factors, and paternal factors. Child-related variables included sex, birth order, and basic immunization status. Maternal/perinatal healthcare variables included antenatal care, postnatal care, place of delivery, and maternal tetanus immunization during pregnancy. Maternal sociodemographic variables included maternal age at delivery, education, employment, health insurance, smoking status, and media access. Household/contextual variables included household size, wealth index, religion of the household head, place of residence, region, and decision-maker for child healthcare. Paternal variables included paternal age, education, employment, health insurance, smoking status, and media access. The complete list of the 28 candidate variables, grouped by domain, is provided in Supplementary Table S1.

Several continuous or multi-category variables were recoded into analytically meaningful categories based on prior studies and the distribution of the data. Socioeconomic status was assessed using the household wealth index, which was derived from principal component analysis (PCA) of household assets and living conditions and categorized into quintiles ranging from the poorest to the richest. Basic immunization status was classified as complete or incomplete according to the child’s receipt of routine basic childhood immunizations recorded in IFLS-5. The specific immunizations included in this definition were adopted from a previous IFLS-based study (14) and comprised BCG, OPV, DPT, measles, and hepatitis B vaccines.

Religion of the household head requires explicit framing. It was recorded in IFLS-5 as the stated affiliation of the household head and was collapsed into Islam versus non-Islam because the individual non-Islam categories were too small to support stable estimation. This variable is treated throughout as a sociocultural and structural marker, indexing the regional, ethnic, community, and health-service-environment differences with which religious affiliation is strongly correlated in Indonesia, and not as a measure of religious belief, religiosity, denominational teaching, or any doctrinal position on vaccination, none of which IFLS-5 records.

### Statistical analysis

Descriptive statistics were used to summarize participant characteristics and rotavirus vaccine uptake. Categorical variables were presented as frequencies and percentages, and the proportion vaccinated was also examined by single year of child age. Multivariable analysis was performed using hierarchical binary logistic regression, with rotavirus vaccine uptake (yes/no) as the dependent variable. Variables were entered in conceptual blocks ordered from the most proximal child-level characteristics to progressively more distal family and contextual characteristics, so that changes in associations could be observed as broader context was introduced. This ordering reflects a conceptual framework in which some variables (for example, complete basic immunization and postnatal care) are best understood as markers of prior engagement with health services rather than as direct causal determinants of rotavirus vaccination. Model 1 included child-related factors: sex of child, birth order, and basic immunization status. Model 2 additionally included maternal/perinatal healthcare factors: postnatal care, place of delivery, and maternal tetanus immunization during pregnancy. Model 3 additionally included maternal sociodemographic factors: maternal age at delivery, maternal education, maternal employment, maternal health insurance, maternal smoking status, and maternal media access. Model 4 additionally included household/contextual factors: religion of household head, household size, wealth index, place of residence, and region. Model 5 additionally included paternal factors with low missingness: paternal age at survey and paternal media access. Adjusted odds ratios (AORs) and 95% confidence intervals (CIs) were reported, and statistical significance was set at *p* < 0.05.

Because IFLS-5 employs a complex multistage sampling design, the decision of whether to incorporate sampling weights merits explicit justification. The primary aim of this analysis was to estimate conditional associations between child, maternal, and household characteristics and rotavirus vaccine uptake, rather than to generate nationally representative prevalence estimates. Methodological work on weighted regression indicates that sampling weights are most important for unbiased population-level descriptive estimates, whereas for model-based analyses of conditional associations, weighting is not always necessary once the substantive design variables are included as covariates in the model; under a correctly specified model, unweighted estimation can in fact be more efficient, because weighting can increase the variance of regression coefficients without a corresponding gain in the precision of the associations of interest (15). Because region, place of residence, and household wealth—three of the principal dimensions underlying the IFLS sampling design—were included directly as covariates in the hierarchical models, design-related sources of variation were substantially, although not completely, accounted for within the regression itself. A model-based (unweighted) approach was therefore adopted as the primary analytic strategy, consistent with the study’s associational rather than descriptive objective; as reported below, this decision was further supported by a sensitivity analysis using household-clustered standard errors.

To limit the loss of observations from missing data, the primary hierarchical analysis was restricted to variables with no more than 10% missingness (16, 17). Accordingly, the primary models included child age, sex of child, birth order, basic immunization status, postnatal care, place of delivery, maternal tetanus immunization during pregnancy, maternal age at delivery, maternal education, maternal employment, maternal health insurance, maternal smoking status, maternal media access, religion of household head, household size, wealth index, place of residence, region, paternal age at survey, and paternal media access. Six variables exceeded this threshold and were excluded from the primary models: child health insurance, decision-maker for child healthcare, paternal health insurance, paternal smoking status, paternal education, and paternal employment. Maternal age at survey was also excluded because of conceptual overlap with maternal age at delivery. The extent of missingness for each variable is reported in Table 1, and the resulting inclusion and exclusion decisions are summarized in Supplementary Table S1.

**Table 1:** Characteristics of children and households in the study sample (*N* = 3,320).

Characteristic	*n* (%)	Missing, *n* (%)
Child age		0 (0)
1 year	518 (15.6)	
2 years	587 (17.7)	
3 years	708 (21.3)	
4 years	749 (22.6)	
5 years	758 (22.8)	
Sex of child		0 (0)
Female	1,630 (49.1)	
Male	1,690 (50.9)	
Religion of household head		329 (9.9)
Non-Islam	327 (9.8)	
Islam	2,664 (80.2)	
Parental marital status		77 (2.3)
Unmarried	88 (2.7)	
Married	3,155 (95.0)	
Antenatal care		274 (8.3)
No	35 (1.1)	
Yes	3,011 (90.7)	
Postnatal care		322 (9.7)
No	1,746 (52.6)	
Yes	1,252 (37.7)	
Birth order		240 (7.2)
First born	1,887 (56.8)	
Second born and later	1,193 (36.0)	
Place of delivery		277 (8.3)
Home	723 (21.8)	
Health facility	1,309 (39.4)	
Other	1,011 (30.5)	
Region		0 (0)
Java	1,512 (45.5)	
Sumatera	1,012 (30.5)	
Bali and Nusa Tenggara	506 (15.2)	
Kalimantan	138 (4.2)	
Sulawesi	152 (4.6)	
Place of residence		0 (0)
Rural	1,389 (41.8)	
Urban	1,931 (58.2)	
Decision-maker for child healthcare		874 (26.3)
Father	346 (10.4)	
Mother	431 (13.0)	
Both parents	1,669 (50.3)	
Maternal tetanus immunization during pregnancy		328 (9.9)
No	723 (21.8)	
Yes	2,269 (68.3)	
Maternal age at delivery		77 (2.3)
≤20 years	381 (11.5)	
21–30 years	1,888 (56.9)	
>30 years	974 (29.3)	
Child health insurance		2,471 (74.4)
No	709 (21.4)	
Yes	140 (4.2)	
Household size		0 (0)
≤4 persons	1,709 (51.5)	
5–9 persons	1,263 (38.0)	
≥10 persons	348 (10.5)	
Wealth index		5 (0.2)
Poorest	602 (18.1)	
Poorer	557 (16.8)	
Middle	779 (23.5)	
Richer	642 (19.3)	
Richest	735 (22.1)	
Maternal age at survey		77 (2.3)
≤30 years	1,619 (48.8)	
31–40 years	1,429 (43.0)	
>40 years	195 (5.9)	
Maternal education		90 (2.7)
Primary	1,517 (45.7)	
Secondary	1,192 (35.9)	
Tertiary	521 (15.7)	
Maternal employment		79 (2.4)
Unemployed	1,979 (59.6)	
Employed	1,262 (38.0)	
Maternal health insurance		226 (6.8)
No	1,522 (45.8)	
Yes	1,572 (47.3)	
Maternal smoking status		226 (6.8)
Never	3,032 (91.3)	
Ever	62 (1.9)	
Maternal media access		0 (0)
No	205 (6.2)	
Yes	3,115 (93.8)	
Paternal age at survey		330 (9.9)
≤30 years	810 (24.4)	
31–40 years	1,620 (48.8)	
41–50 years	497 (15.0)	
>50 years	63 (1.9)	
Paternal education		355 (10.7)
Primary	1,331 (40.1)	
Secondary	1,189 (35.8)	
Tertiary	445 (13.4)	
Paternal employment		338 (10.2)
Unemployed	222 (6.7)	
Employed	2,760 (83.1)	
Paternal health insurance		860 (25.9)
No	1,194 (36.0)	
Yes	1,266 (38.1)	
Paternal smoking status		860 (25.9)
Never	497 (15.0)	
Ever	1,963 (59.1)	
Paternal media access		0 (0)
No	143 (4.3)	
Yes	3,177 (95.7)	
Basic immunization status		0 (0)
Incomplete	781 (23.5)	
Complete	2,539 (76.5)	

Child age is reported in completed years at the time of survey; the analytic sample comprised children aged 1–5 years.

Complete-case analysis rather than multiple imputation was retained as the primary strategy, for three reasons, and multiple imputation was then performed as a sensitivity analysis so that the two approaches could be compared directly. First, the validity of complete-case analysis in a regression setting rests on a weaker assumption than the one required by multiple imputation. Multiple imputation yields unbiased estimates only if data are missing at random (MAR) conditional on the variables entered into the imputation model. Complete-case estimates of regression coefficients, by contrast, are asymptotically unbiased whenever the probability of being a complete case is independent of the outcome given the covariates in the analysis model, a condition that holds under MAR and also under a broad class of missing-not-at-random mechanisms in which missingness depends on the covariates themselves, however strongly (18, 19). This distinction is directly relevant here, because missingness was confined entirely to covariates: rotavirus vaccination status defined the analytic sample and was complete by construction. Second, that condition was testable and was tested formally, as described below, rather than being assumed; the children included in and excluded from the fully adjusted model were also compared directly on observed characteristics (Supplementary Table S2). Third, the variables with the most severe missingness are those for which MAR is least tenable: paternal education, employment, health insurance, and smoking status are missing largely because no father was resident in, interviewed in, or linkable to the household roster, and decision-maker for child healthcare is missing through item non-response in a module administered to only a subset of respondents. Missingness of this kind is structural and may depend on the unobserved values themselves, so imputing these variables would rest on an assumption that cannot be checked.

We nevertheless recognize that complete-case analysis discards information, reduces precision, and can be biased if its identifying condition fails, and that these concerns cannot be settled by argument alone. Four additional analyses were therefore specified. (i) The complete-case condition was tested directly by regressing an indicator of complete-case status on the outcome together with all fully observed covariates; under the condition required for unbiasedness, the outcome should not predict complete-case status. (ii) The fully adjusted model was refitted using inverse-probability weights, each complete case being weighted by the reciprocal of its estimated probability of being a complete case given the fully observed covariates and the outcome. This provides an estimator that corrects for selection on observed variables and is distinct from both unweighted complete-case analysis and imputation. (iii) Because the test in (i) can bound but not exclude modest outcome-dependent missingness, a tipping-point analysis was performed in which outcome-dependent selection was deliberately imposed at a range of magnitudes (assumed odds ratios for the outcome predicting completeness from 0.40 to 1.50, the lower bound being considerably stronger than the value actually observed), the complete cases reweighted accordingly, and the fully adjusted model refitted at each value. (iv) Multiple imputation by chained equations was performed on all 3,320 children, generating m = 20 imputed datasets with 10 cycles of fully conditional specification. Binary and multi-category variables were imputed using logistic and multinomial logistic models with a weak ridge penalty to prevent separation in sparse cells; the outcome was included as a predictor in every imputation model, as required for valid imputation of covariates; and parameter uncertainty was induced by fitting each conditional model to a bootstrap resample of the observed rows. The substantive model was fitted within each imputed dataset and the results pooled using Rubin’s rules, with Barnard-Rubin adjusted degrees of freedom (20–22). Firth penalized logistic regression was additionally fitted as described below.

These analyses have complementary purposes, and we distinguish them deliberately. Analyses (i) to (iii) interrogate whether the complete-case estimates are distorted by selection on observed variables; they operate within the complete-case sample and, by construction, cannot recover information carried by the 634 excluded children. Multiple imputation is the only analysis that uses the whole sample, and it is therefore the decisive check on whether an association depends on the complete-case restriction itself. We accordingly adopt the following rule, specified before the results were examined and applied without exception: where multiple imputation disagrees with the complete-case model, we defer to multiple imputation and report the association as not robust, irrespective of which analysis is designated primary. Retaining complete-case analysis as the primary specification is a statement about which estimator we consider best identified for this data structure; it is not a license to prefer its results.

During model diagnostics, two variables produced unstable parameter estimates characterized by implausibly large coefficients and standard errors: antenatal care and parental marital status. Both had highly skewed distributions with very few children in the reference category (Table 1), and within the complete-case sample the problem proved to be quasi-complete separation rather than mere sparseness, so that maximum-likelihood estimation did not yield interpretable coefficients; the relevant cell counts and divergent estimates are reported in the Results. In such settings maximum-likelihood coefficients are biased away from the null, standard errors are inflated, and Wald-based confidence intervals have poor coverage (23, 24). Both variables were therefore excluded from the primary maximum-likelihood models. Rather than leaving them out altogether, we refitted the fully adjusted model using Firth penalized likelihood, which applies a Jeffreys-prior penalty that removes the first-order bias of maximum likelihood and yields finite estimates under separation (25, 26). Antenatal care and parental marital status were restored to this model, and inference used profile penalized-likelihood confidence intervals and penalized likelihood-ratio tests rather than Wald statistics, which behave poorly with sparse data. The same model provides penalized estimates for the sparse categories retained in the primary models (maternal smoking, paternal age above 50 years, and absence of maternal media access), and the religion-by-region interaction reported below was tested by penalized likelihood-ratio test for the same reason.

Multicollinearity in the fully adjusted model was assessed using generalized variance inflation factors (GVIF), which appropriately account for categorical predictors with more than one degree of freedom by using [GVIF^(1/(2·Df))]^2^, squared for comparability with the conventional VIF scale. Across all 19 predictors, the mean adjusted GVIF was 1.16 and the maximum was 1.48 (maternal media access), both well below conventional thresholds of concern (5 or 10), indicating no meaningful multicollinearity; the full variable-by-variable results are provided in Supplementary Table S3.

Children included in the fully adjusted model were compared with those excluded because of missing data on key observed characteristics (Supplementary Table S2), and sensitivity analyses were conducted by adding each excluded high-missingness variable individually to the fully adjusted model (Model 5), namely paternal education, paternal employment, paternal health insurance, paternal smoking status, and decision-maker for child healthcare. To verify that non-independence among children from the same household did not affect inference, the fully adjusted model was also refitted with standard errors clustered on household (Supplementary Table S4).

Child age at interview was included as a continuous covariate, in single completed years, in all five hierarchical models. Because IFLS-5 was a single-wave, cross-sectional survey, age at interview and birth cohort are a deterministic function of one another (birth year ≈ survey year − age in completed years); the two cannot be separated within these data, and a model containing single-year age indicators is therefore mathematically equivalent to a model containing birth-cohort indicators. To test whether the linear specification obscured a non-linear or cohort-specific pattern, age was additionally modeled as a set of single-year indicators, which we report and interpret as a birth-cohort analysis (Supplementary Table S5); the two specifications were compared by likelihood-ratio test, and uptake by single year of age was also compared using a chi-squared test. To aid public-health interpretation alongside the odds ratios, model-based predicted probabilities of uptake were estimated for selected key variables. Because the objective was to estimate conditional associations rather than population-level prevalence, a model-based (unweighted) approach was adopted, and the principal dimensions underlying the survey design (region, place of residence, and household wealth) were included directly as covariates. Model performance was evaluated using the Hosmer-Lemeshow goodness-of-fit test, the area under the receiver operating characteristic curve (AUC), and pseudo-*R*^2^ statistics (McFadden, Cox-Snell, and Nagelkerke).

Finally, because religion of the household head is interpreted as a contextual marker rather than as a causal factor, three descriptive analyses were specified to characterize what the variable actually indexes in these data: the joint distribution of religion and region; the sequential change in the religion coefficient as region, place of residence, wealth, and maternal education were added; and region-stratified associations together with a formal test for effect modification of religion by region (Supplementary Table S6).

### Ethics approval

This study analyzed publicly accessible, de-identified secondary data from the fifth wave of the Indonesia Family Life Survey (IFLS-5). The original IFLS data collection was reviewed and approved by the Institutional Review Boards (IRBs) of the RAND Corporation in the United States and of Universitas Gadjah Mada in Indonesia, under protocol approval number s0064-06-01-CR01. Informed consent was obtained from all participants. As the present study was limited to the secondary analysis of anonymized existing data and involved no recruitment of, direct interaction with, or additional data collection from human participants, separate ethical approval was not sought. This determination is consistent with the 2022 Standard Operating Procedures of the Research Ethics Committee at Universitas Padjadjaran, Indonesia (No. POB/08/KEP), which indicate that studies using pre-existing or publicly accessible data, documents, and diagnostic or pathological materials may be exempt from ethical review when individuals’ identities and confidentiality are adequately protected. The de-identified IFLS-5 data analyzed here were obtained from the RAND IFLS data repository under the standard data user agreement, and no attempt was made to re-identify any individual respondent.

## Results

### Participant characteristics

Rotavirus vaccination status was available for 3,320 children, of whom 287 had received at least one dose of rotavirus vaccine, corresponding to an uptake of 8.64%. All children in the analytic sample were aged 1–5 years, and uptake was broadly similar across single years of age, ranging from 7.1% at 1 year to 10.7% at 2 years with no clear trend with age (Tables 1 and 2). The sample was nearly evenly distributed by sex, with boys accounting for 50.9% of participants. Most children lived in Islam-headed households (80.2%), and almost half resided in Java (45.5%), while 58.2% lived in urban areas. Household size was generally modest, with 51.5% of children living in households of four or fewer members. Maternal and paternal media access was high, at 93.8 and 95.7%, respectively. Missing data were low for most core variables, although several variables considered for later modeling had substantial missingness, particularly child health insurance, decision-maker for child healthcare, paternal health insurance, and paternal smoking status (Table 1).

Comparison of the 2,686 children included in the fully adjusted model with the 634 children excluded because of missing data showed broadly similar distributions (Supplementary Table S2). Reported uptake was 8.3% among included children and 10.3% among excluded children (*p* = 0.13), and the two groups were comparable with respect to child sex, religion of household head, place of residence, and wealth index; excluded children were more likely to be from Java and had a slightly lower proportion with complete basic immunization (72.7% versus 77.4%). These modest differences suggest that the complete-case approach is unlikely to have introduced major selection bias, although it cannot be entirely excluded.

### Bivariate associations with rotavirus vaccine uptake

In the bivariate analysis, several variables were associated with rotavirus vaccine uptake. Children from Islam-headed households had lower odds of receiving rotavirus vaccine than those from non-Islam-headed households (OR 0.45, 95% CI 0.32–0.63; *p* < 0.001). Uptake was also higher among children whose mothers had received postnatal care (OR 1.37, 95% CI 1.05–1.77; *p* = 0.018) and among those with complete basic immunization (OR 2.71, 95% CI 1.85–3.98; *p* < 0.001). Compared with children living in Java, those living in Sulawesi had higher odds of rotavirus vaccination (OR 2.46, 95% CI 1.58–3.84; p < 0.001). Decision-maker for child healthcare and paternal smoking status were also associated with uptake in the unadjusted analysis, whereas many other variables, including child sex, birth order, place of delivery, household size, wealth index, and place of residence, were not significantly associated (Table 2).

**Table 2:** Bivariate association between participant characteristics and rotavirus vaccine uptake.

Characteristic	Rotavirus uptake (*n* = 3,320)		
	Yes (*n* = 287), *n* (%)ᵃ	*p*-value (*χ*^2^ test)	Unadjusted OR (95% CI)
Child age			
1 year	37 (12.9)	Reference	1
2 years	63 (22.0)	0.039*	1.56 (1.02–2.39)
3 years	61 (21.3)	0.348	1.23 (0.80–1.87)
4 years	62 (21.6)	0.460	1.17 (0.77–1.79)
5 years	64 (22.3)	0.399	1.20 (0.79–1.83)
Sex of child			
Female	133 (46.3)	Reference	1
Male	154 (53.7)	0.329	1.13 (0.89–1.44)
Religion of household head			
Non-Islam	51 (20.0)	Reference	1
Islam	204 (80.0)	<0.001*	0.45 (0.32–0.63)
Parental marital status			
Unmarried	11 (3.9)	Reference	1
Married	269 (96.1)	0.190	0.65 (0.34–1.24)
Antenatal care			
No	1 (0.4)	Reference	1
Yes	258 (99.6)	0.228	3.18 (0.43–23.37)
Postnatal care			
No	128 (51.2)	Reference	1
Yes	122 (48.8)	0.018*	1.37 (1.05–1.77)
Birth order			
First born	158 (60.1)	Reference	1
Second born and later	105 (39.9)	0.679	1.06 (0.82–1.37)
Place of delivery			
Home	55 (21.2)	Reference	1
Health facility	115 (44.4)	0.359	1.17 (0.84–1.63)
Other	89 (34.4)	0.374	1.17 (0.83–1.67)
Region			
Java	132 (46.0)	Reference	1
Sumatera	77 (26.8)	0.317	0.86 (0.64–1.15)
Bali and Nusa Tenggara	37 (12.9)	0.320	0.82 (0.56–1.21)
Kalimantan	12 (4.2)	0.989	0.99 (0.54–1.85)
Sulawesi	29 (10.1)	<0.001*	2.46 (1.58–3.84)
Place of residence			
Rural	120 (41.8)	Reference	1
Urban	167 (58.2)	0.993	1.00 (0.78–1.28)
Decision-maker for child healthcare			
Father	37 (18.2)	Reference	1
Mother	25 (12.3)	0.014*	0.51 (0.30–0.87)
Both parents	141 (69.5)	0.181	0.77 (0.53–1.13)
Maternal tetanus immunization during pregnancy			
No	54 (21.3)	Reference	1
Yes	200 (78.7)	0.258	1.20 (0.88–1.64)
Maternal age at delivery			
≤20 years	33 (11.8)	Reference	1
21–30 years	144 (51.4)	0.493	0.87 (0.59–1.30)
>30 years	103 (36.8)	0.293	1.25 (0.83–1.88)
Child health insurance			
No	66 (86.8)	Reference	1
Yes	10 (13.2)	0.412	0.75 (0.38–1.50)
Household size			
≤4 persons	146 (50.9)	Reference	1
5–9 persons	102 (35.5)	0.649	0.94 (0.72–1.22)
≥10 persons	39 (13.6)	0.115	1.35 (0.93–1.96)
Wealth index			
Poorest	48 (16.7)	Reference	1
Poorer	47 (16.4)	0.773	1.06 (0.70–1.62)
Middle	60 (20.9)	0.852	0.96 (0.65–1.43)
Richer	60 (20.9)	0.391	1.19 (0.80–1.77)
Richest	72 (25.1)	0.247	1.25 (0.86–1.84)
Maternal age at survey			
≤30 years	126 (45.0)	Reference	1
31–40 years	131 (46.8)	0.170	1.20 (0.93–1.54)
>40 years	23 (8.2)	0.056	1.58 (0.99–2.54)
Maternal education			
Primary	131 (47.0)	Reference	1
Secondary	102 (36.6)	0.942	0.99 (0.75–1.29)
Tertiary	46 (16.5)	0.892	1.02 (0.72–1.46)
Maternal employment			
Unemployed	170 (60.7)	Reference	1
Employed	110 (39.3)	0.901	1.02 (0.79–1.30)
Maternal health insurance			
No	122 (46.9)	Reference	1
Yes	138 (53.1)	0.444	1.10 (0.86–1.43)
Maternal smoking status			
Never	252 (96.9)	Reference	1
Ever	8 (3.1)	0.197	1.64 (0.77–3.47)
Maternal media access			
No	16 (5.6)	Reference	1
Yes	271 (94.4)	0.659	1.13 (0.67–1.90)
Paternal age at survey			
≤30 years	67 (26.3)	Reference	1
31–40 years	143 (56.1)	0.646	1.07 (0.80–1.45)
41–50 years	38 (14.9)	0.686	0.92 (0.61–1.39)
>50 years	7 (2.7)	0.438	1.39 (0.61–3.16)
Paternal education			
Primary	103 (40.7)	Reference	1
Secondary	112 (44.3)	0.132	1.24 (0.94–1.64)
Tertiary	38 (15.0)	0.589	1.11 (0.76–1.64)
Paternal employment			
Unemployed	18 (7.1)	Reference	1
Employed	237 (92.9)	0.806	1.06 (0.65–1.76)
Paternal health insurance			
No	87 (42.9)	Reference	1
Yes	116 (57.1)	0.091	1.28 (0.96–1.72)
Paternal smoking status			
Never	59 (29.1)	Reference	1
Ever	144 (70.9)	0.001*	0.58 (0.43–0.81)
Paternal media access			
No	9 (3.1)	Reference	1
Yes	278 (96.9)	0.306	1.43 (0.72–2.84)
Basic immunization status			
Incomplete	31 (10.8)	Reference	1
Complete	256 (89.2)	<0.001*	2.71 (1.85–3.98)

^a^Total number varies between categories due to missing data.

*Significant (*p* < 0.05).

### Hierarchical multivariable models

In the hierarchical logistic regression analysis, complete basic immunization remained positively associated with rotavirus vaccine uptake across all five models. Postnatal care was also positively associated with uptake once maternal and perinatal healthcare variables were introduced. In the fully adjusted model, based on 2,686 complete cases, complete basic immunization (AOR 2.71, 95% CI 1.73–4.24) and receipt of postnatal care (AOR 1.57, 95% CI 1.17–2.11) were associated with higher odds of uptake. Maternal age at delivery above 30 years was associated with higher uptake than maternal age at delivery of 20 years or younger (AOR 1.85, 95% CI 1.02–3.37), while secondary maternal education was associated with lower uptake than primary education (AOR 0.69, 95% CI 0.49–0.97). Children from Islam-headed households had lower odds of vaccination than those from non-Islam-headed households (AOR 0.31, 95% CI 0.21–0.47). Compared with Java, uptake was lower in Bali and Nusa Tenggara (AOR 0.51, 95% CI 0.31–0.84) and higher in Sulawesi (AOR 2.81, 95% CI 1.64–4.81). Among paternal characteristics, only paternal age 41–50 years remained associated with lower uptake compared with paternal age ≤30 years (AOR 0.45, 95% CI 0.25–0.81). Male children also showed higher odds of uptake in the fully adjusted model (AOR 1.34, 95% CI 1.01–1.79); however, this association emerged only after later adjustment and was of borderline significance, and is therefore interpreted with caution. Other variables included in the later models, such as place of delivery, maternal tetanus immunization during pregnancy, maternal employment, maternal health insurance, household size, wealth index, place of residence, and paternal media access, were not independently associated with uptake after adjustment (Table 3).

**Table 3:** Factors associated with rotavirus vaccine uptake based on hierarchical logistic regression models.

Characteristic	AOR (95% CI)				
	Model 1 (*n* = 3,080)	Model 2 (*n* = 2,959)	Model 3 (*n* = 2,942)	Model 4 (*n* = 2,687)	Model 5 (*n* = 2,686)
Child age, per year (continuous)	0.98 (0.89–1.07)	0.99 (0.89–1.09)	0.98 (0.89–1.08)	0.98 (0.88–1.09)	1.02 (0.91–1.13)
Sex of child (Ref Female)					
Male	1.22 (0.94–1.57)	1.22 (0.94–1.59)	1.24 (0.95–1.61)	1.35 (1.02–1.80)*	1.34 (1.01–1.79)*
Birth order (Ref First born)					
Second born and later	1.07 (0.82–1.40)	1.11 (0.85–1.45)	1.04 (0.78–1.39)	0.99 (0.72–1.35)	1.03 (0.75–1.42)
Basic immunization status (Ref Incomplete)					
Complete	2.93 (1.94–4.43)*	2.64 (1.74–4.01)*	2.80 (1.83–4.29)*	2.66 (1.71–4.16)*	2.71 (1.73–4.24)*
Postnatal care (Ref No)					
Yes		1.32 (1.01–1.72)*	1.35 (1.04–1.77)*	1.57 (1.17–2.11)*	1.57 (1.17–2.11)*
Place of delivery (Ref Home)					
Health facility		1.11 (0.78–1.57)	1.13 (0.78–1.62)	1.25 (0.82–1.89)	1.26 (0.83–1.91)
Other		1.17 (0.82–1.67)	1.20 (0.83–1.72)	1.31 (0.86–1.98)	1.33 (0.88–2.03)
Maternal tetanus immunization during pregnancy (Ref No)					
Yes		1.14 (0.83–1.57)	1.11 (0.81–1.53)	1.24 (0.87–1.76)	1.28 (0.89–1.82)
Maternal age at delivery (Ref ≤ 20 years)					
21–30 years			0.81 (0.52–1.25)	0.80 (0.50–1.28)	0.93 (0.56–1.53)
>30 years			1.19 (0.74–1.91)	1.27 (0.75–2.13)	1.85 (1.02–3.37)*
Maternal education (Ref Primary)					
Secondary			0.84 (0.63–1.13)	0.73 (0.52–1.02)	0.69 (0.49–0.97)*
Tertiary			0.83 (0.55–1.25)	0.70 (0.44–1.11)	0.67 (0.42–1.06)
Maternal employment (Ref Unemployed)					
Employed			1.04 (0.79–1.38)	1.02 (0.75–1.38)	1.02 (0.76–1.39)
Maternal health insurance (Ref No)					
Yes			1.07 (0.82–1.40)	1.01 (0.76–1.35)	1.01 (0.75–1.35)
Maternal smoking status (Ref Never)					
Ever			1.76 (0.78–4.00)	1.61 (0.64–4.06)	1.70 (0.67–4.30)
Maternal media access (Ref No)					
Yes			1.03 (0.58–1.84)	0.97 (0.52–1.81)	0.95 (0.47–1.93)
Religion of household head (Ref Non-Islam)					
Islam				0.32 (0.21–0.48)*	0.31 (0.21–0.47)*
Household size (Ref ≤ 4 persons)					
5–9 persons				0.76 (0.54–1.06)	0.81 (0.57–1.14)
≥10 persons				1.13 (0.70–1.83)	1.22 (0.76–1.98)
Wealth index (Ref Poorest)					
Poorer				1.19 (0.72–1.97)	1.21 (0.73–2.01)
Middle				1.15 (0.71–1.85)	1.15 (0.72–1.86)
Richer				1.36 (0.83–2.22)	1.38 (0.85–2.26)
Richest				1.37 (0.85–2.22)	1.39 (0.86–2.25)
Place of residence (Ref Rural)					
Urban				0.95 (0.69–1.30)	0.94 (0.68–1.29)
Region (Ref Java)					
Sumatera				0.80 (0.55–1.17)	0.79 (0.54–1.15)
Bali and Nusa Tenggara				0.53 (0.32–0.87)*	0.51 (0.31–0.84)*
Kalimantan				1.33 (0.69–2.57)	1.33 (0.69–2.56)
Sulawesi				2.83 (1.65–4.84)*	2.81 (1.64–4.81)*
Paternal age at survey (Ref ≤ 30 years)					
31–40 years					0.76 (0.52–1.11)
41–50 years					0.45 (0.25–0.81)*
>50 years					0.45 (0.14–1.45)
Paternal media access (Ref No)					
Yes					1.07 (0.45–2.58)

*Significant (*p* < 0.05). Model performance (fully adjusted model): AUC = 0.70; Hosmer-Lemeshow *χ*^2^ = 6.19, df = 8, *p* = 0.626; pseudo-*R*2 (Nagelkerke) = 0.095.

To support interpretation, model-based predicted probabilities of uptake were estimated for the most consistent correlates, holding other covariates at their observed values. Predicted uptake was 9.6% for children with complete basic immunization versus 3.9% for those with incomplete immunization, 10.2% versus 6.9% for children whose mothers did versus did not receive postnatal care, and 18.9% versus 7.2% for children from non-Islam-headed versus Islam-headed households. These differences are consistent with the odds ratios and underline the public-health relevance of prior health-service contact and contextual factors.

Child age was included as a continuous covariate in all five hierarchical models and was not associated with rotavirus vaccine uptake in any of them (Model 5 AOR 1.02 per year, 95% CI 0.91–1.13; Table 3); the per-year estimate was likewise null in the unadjusted analysis (crude OR 0.99, 95% CI 0.91–1.09), and uptake did not differ significantly across single years of age (7.1 to 10.7%; *χ*^2^ = 4.89, df = 4, *p* = 0.30). Because age at interview and year of birth are equivalent in a single-wave survey, these models can be read directly as a birth-cohort analysis. The children studied were born between approximately 2009 and 2014, so their eligibility windows fell between approximately 2009 and 2015, entirely within the pre-introduction period during which the vaccine was available only through private, self-paid provision. No child was outside the age of eligibility during the period in which the vaccine was obtainable, so differential programmatic eligibility cannot account for the observed variation in uptake.

Respecifying age as single-year birth-cohort indicators did not produce a monotonic cohort gradient (Supplementary Table S5). Relative to the youngest cohort (children aged 1 year at interview, born approximately 2013–14), only the 2012–13 cohort differed significantly (AOR 1.95, 95% CI 1.18–3.22, *p* = 0.009); the estimates for the three older cohorts declined steadily toward the reference (AOR 1.64, 1.48 and 1.38 respectively, all non-significant), and the overall likelihood-ratio test comparing the categorical with the linear specification was of borderline significance (*χ*^2^ = 7.67, df = 3, *p* = 0.053). We do not interpret this as evidence of a cohort effect on uptake: the pattern is non-monotonic, it is driven by a single contrast, it is not supported by the unadjusted comparison across age groups, and it is not what a secular increase in private-market availability would predict. Most importantly for the present purpose, the choice of specification was immaterial to every other result: no adjusted odds ratio in Model 5 changed by more than 0.04 when birth-cohort indicators replaced linear age. The reported associations are therefore explained neither by the age composition of the sample nor by cohort differences in vaccine eligibility, in elapsed time since the eligibility window, or in the maturity of the private market over the 2009–2015 period.

The fully adjusted model showed acceptable discrimination and adequate calibration. The area under the receiver operating characteristic curve (AUC) was 0.70, indicating modest-to-acceptable ability to distinguish children who received rotavirus vaccine from those who did not, consistent with a model of socio-behavioral correlates rather than a clinical prediction tool. The Hosmer-Lemeshow goodness-of-fit test showed no evidence of miscalibration (*χ*^2^ = 6.19, df = 8, *p* = 0.626). Pseudo-*R*^2^ values were modest (McFadden *R*^2^ = 0.074; Cox-Snell *R*^2^ = 0.041; Nagelkerke *R*^2^ = 0.095), consistent with the low prevalence of the outcome (8.6%) and with a model designed to identify correlates rather than to maximize predictive accuracy.

### Sensitivity analyses

Sensitivity analyses showed that the main findings were generally stable when excluded high-missing variables were added individually to the fully adjusted model. The associations for complete basic immunization, postnatal care, religion of household head, regional variation, and paternal age 41–50 years changed little in direction or magnitude across sensitivity models. In contrast, the associations for male sex and maternal age at delivery above 30 years were less consistent, with loss of statistical significance in some models despite similar point estimates. These changes were accompanied by reductions in sample size in several sensitivity models, suggesting lower precision rather than substantial reversal of effect estimates (Table 4). Refitting the fully adjusted model with standard errors clustered on household, to allow for non-independence among siblings, produced materially unchanged estimates and confidence intervals, indicating that the inferences were not sensitive to within-household clustering (Supplementary Table S4).

**Table 4:** Sensitivity analyses of factors associated with rotavirus vaccine uptake.

Model (added variable)	N	AOR (95% CI)								
		Male sex	Complete immunization	Postnatal care	Maternal age at delivery >30 years	Secondary maternal education	Islam household head	Region: Bali and Nusa Tenggara	Region: Sulawesi	Paternal age 41–50
Main model	2,686	1.34 (1.01–1.79)*	2.71 (1.73–4.24)*	1.57 (1.17–2.11)*	1.85 (1.02–3.37)*	0.69 (0.49–0.97)*	0.31 (0.21–0.47)*	0.51 (0.31–0.84)*	2.81 (1.64–4.81)*	0.45 (0.25–0.81)*
Sensitivity 1 (Decision-maker for child healthcare)	2,255	1.48 (1.07–2.04)*	3.43 (2.02–5.82)*	1.59 (1.15–2.21)*	1.88 (0.98–3.61)	0.67 (0.46–0.98)*	0.24 (0.15–0.39)*	0.46 (0.26–0.79)*	2.19 (1.16–4.16)*	0.37 (0.20–0.71)*
Sensitivity 2 (Paternal education)	2,666	1.32 (0.99–1.76)	2.82 (1.78–4.45)*	1.59 (1.18–2.13)*	1.93 (1.05–3.53)*	0.63 (0.44–0.91)*	0.31 (0.20–0.47)*	0.50 (0.30–0.83)*	2.66 (1.54–4.61)*	0.45 (0.25–0.80)*
Sensitivity 3 (Paternal employment)	2,681	1.34 (1.01–1.79)*	2.72 (1.74–4.25)*	1.56 (1.17–2.10)*	1.86 (1.02–3.40)*	0.69 (0.49–0.97)*	0.31 (0.21–0.47)*	0.51 (0.31–0.84)*	2.79 (1.63–4.78)*	0.45 (0.25–0.80)*
Sensitivity 4 (Paternal health insurance)	2,245	1.45 (1.05–1.99)*	3.52 (2.07–5.98)*	1.59 (1.14–2.20)*	1.86 (0.97–3.58)	0.66 (0.45–0.97)*	0.25 (0.16–0.40)*	0.46 (0.27–0.80)*	2.16 (1.14–4.07)*	0.37 (0.19–0.70)*
Sensitivity 5 (Paternal smoking status)	2,245	1.42 (1.03–1.96)*	3.44 (2.02–5.83)*	1.55 (1.11–2.15)*	1.84 (0.96–3.54)	0.67 (0.46–0.98)*	0.25 (0.16–0.40)*	0.47 (0.27–0.82)*	2.23 (1.18–4.20)*	0.37 (0.19–0.71)*

Sensitivity analyses were performed by adding excluded variables individually to the fully adjusted model. Results were considered robust when the direction and magnitude of the adjusted odds ratios remained similar across models, regardless of minor changes in statistical significance. *Significant (*p* < 0.05).

The fully adjusted model was fitted to 2,686 of 3,320 children (80.9%). Incompleteness was largely structural rather than outcome-related: 52.1% of the 634 excluded records were missing paternal age, and a model for complete-case status using all fully observed covariates explained little variation (pseudo-*R*^2^ = 0.049), with region and household size the strongest predictors. Testing the assumption under which complete-case regression is unbiased, the outcome was not a significant predictor of complete-case status after adjustment for all fully observed covariates (OR 0.77, 95% CI 0.57–1.04, *p* = 0.09), consistent with the unadjusted comparison of uptake between included and excluded children (8.3% versus 10.3%, *p* = 0.13). The point estimate nonetheless lies below unity, so the condition is supported rather than established, and two further analyses addressed this directly.

Inverse-probability weighting of the complete cases produced almost identical estimates: complete basic immunization AOR 2.60 (95% CI 1.76–3.83), postnatal care 1.56 (1.20–2.02), Islam-headed household 0.32 (0.23–0.46), Bali and Nusa Tenggara 0.51 (0.34–0.78) and Sulawesi 2.89 (1.83–4.59). In a tipping-point analysis, all five principal associations retained their direction, magnitude and statistical significance across the entire imposed range (Supplementary Table S7). Selection on observed variables of any magnitude plausible for these data is therefore not sufficient to generate the principal findings.

Multiple imputation addressed the different question of whether any association depends on the complete-case restriction itself. Under multiple imputation using all 3,320 children (m = 20; median fraction of missing information 0.02), the four principal findings were confirmed: complete basic immunization (AOR 2.78, 95% CI 1.88–4.11), postnatal care (AOR 1.40, 95% CI 1.06–1.85), Islam-headed household (AOR 0.32, 95% CI 0.22–0.48), and region (Bali and Nusa Tenggara AOR 0.61, 95% CI 0.39–0.93; Sulawesi AOR 2.44, 95% CI 1.51–3.96). Four weaker associations did not persist: male sex (AOR 1.13, 95% CI 0.88–1.45), secondary maternal education (AOR 0.82, 95% CI 0.61–1.11), maternal age at delivery above 30 years (AOR 1.56, 95% CI 0.90–2.70), and paternal age 41–50 years (AOR 0.60, 95% CI 0.34–1.04), all of which lost statistical significance and moved toward the null (Supplementary Table S8). This divergence is a selection effect rather than an artifact of imputation: refitting a reduced model containing only the fully observed variables, with no imputation of any kind, reproduces the same shift between the full sample and the complete-case subset (for male sex, OR 1.14 in all 3,320 children versus 1.37 in the 2,686 complete cases). Consistently with this, all four associations persisted under inverse-probability weighting and across the entire tipping-point range (Supplementary Table S7), which is what would be expected: reweighting the complete cases cannot recover information carried by the excluded children, and only multiple imputation uses them. Applying the rule specified in the Methods, we therefore defer to multiple imputation, treat these four associations as not robust, and do not interpret them further.

Within the complete-case sample the two excluded variables showed quasi-complete separation: none of the 9 children without antenatal care and none of the 10 children with unmarried parents had received rotavirus vaccine, so maximum-likelihood coefficients diverged (antenatal care *β* = 28.7, standard error 1.96 × 10^6^; parental marital status β = 26.0, standard error 4.56 × 10^5^). Under Firth penalized likelihood both variables could be restored to the fully adjusted model and both were null: antenatal care AOR 1.69 (95% CI 0.18–10.07, *p* = 0.71) and married parents AOR 2.10 (95% CI 0.26–9.95, *p* = 0.57). Restoring them left the principal findings essentially unchanged: complete basic immunization 2.71 to 2.62, postnatal care 1.57 to 1.55, Islam-headed household 0.31 to 0.32, Bali and Nusa Tenggara 0.51 to 0.52, and Sulawesi 2.81 to 2.76, a maximum change of 3.3% (Supplementary Table S9). The sparse categories retained in the primary models shrank toward the null under penalization, as expected, and remained non-significant (maternal smoking AOR 1.76, 95% CI 0.66–4.03; paternal age above 50 years AOR 0.49, 95% CI 0.14–1.37).

Religious affiliation was strongly patterned by geography (Supplementary Table S6). Non-Islam-headed households made up 28.6% of the sample in Bali and Nusa Tenggara and 15.2% in Sumatera, but only 2.7% in Java and 0.8% in Kalimantan; 83.2% of all non-Islam-headed households were located in Sumatera or Bali and Nusa Tenggara, and the contrast is identified by just 301 non-Islam-headed households and 46 vaccinated children in the complete-case sample. The association also differed markedly across regions. In crude region-stratified analyses the odds ratio for Islam-headed households was 0.15 (95% CI 0.07–0.33) in Bali and Nusa Tenggara, 0.17 (95% CI 0.06–0.50) in Sulawesi and 0.44 (95% CI 0.25–0.77) in Sumatera, but was in the opposite direction and highly imprecise in Java (3.46, 95% CI 0.47–25.52, based on 36 non-Islam-headed households) and not estimable in Kalimantan. A test for effect modification confirmed this heterogeneity (penalized likelihood-ratio test, *χ*^2^ = 14.55, df = 3, *p* = 0.002). Notably, the association strengthened rather than attenuated as contextual variables were added, from an unadjusted OR of 0.45 (95% CI 0.32–0.63) to 0.36 after adjustment for region, 0.35 after further adjustment for place of residence and wealth, and 0.31 in the fully adjusted model, indicating that the coefficient is not simply a proxy for the measured dimensions of socioeconomic position.

## Discussion

This study found that rotavirus vaccine uptake among young Indonesian children was low, with 287 of 3,320 children (8.64%) reported to have received at least one dose. In the fully adjusted analysis, the most consistent correlates of uptake were complete basic immunization, postnatal care, religion of household head, and geographic region. These associations remained largely stable in the sensitivity analyses, whereas the associations for male sex and maternal age at delivery above 30 years were less consistent and should be interpreted with greater caution. Overall, the findings suggest that rotavirus vaccine uptake in Indonesia during the IFLS-5 period was influenced not only by family characteristics, but also by prior engagement with health services and by broader sociocultural and geographic context.

The low uptake observed in this study is understandable in light of the policy context at the time of the survey. As noted earlier, rotavirus vaccine had not yet been included in the national immunization program, meaning that parents had to obtain and pay for it out of pocket (9). In this context, uptake likely depended not only on willingness to vaccinate, but also on awareness, affordability, provider recommendation, and local availability (27–29). This may help explain why coverage remained low even though routine childhood immunization was already more established.

The clearest finding in this study was the association between complete basic immunization and rotavirus vaccine uptake. Children who had completed the basic immunization schedule had substantially higher odds of receiving rotavirus vaccine across all hierarchical models, and this pattern remained stable in the sensitivity analyses. This finding suggests that rotavirus vaccination is closely linked to broader engagement with preventive child healthcare. Families who complete routine immunization may be more familiar with vaccination services, more trusting of immunization, or more likely to act on provider advice (30–32). In practical terms, this suggests that efforts to increase rotavirus vaccine uptake may be more effective when integrated into existing immunization contacts rather than delivered as a separate strategy (30, 33).

Postnatal care showed a similarly consistent positive association. Both complete basic immunization and postnatal care are best interpreted as markers of ongoing contact with the health system rather than as direct causes of rotavirus vaccination. Mothers who attend postnatal care may have greater exposure to health information, stronger links to formal care, and more opportunities to receive counseling on child vaccination (34, 35). In this sense, postnatal care may function more as a marker of ongoing engagement with health services than as a single isolated encounter. Taken together, the findings for complete basic immunization and postnatal care suggest that rotavirus vaccine uptake is partly embedded in a broader pattern of preventive care use.

Religion of the household head was, together with geographic region, among the most prominent contextual correlates in the final model, and the association survived both multiple imputation and penalization. Children in Islam-headed households had markedly lower observed odds of rotavirus vaccine uptake than those in non-Islam-headed households. We interpret this variable as a sociocultural and structural marker rather than as a causal determinant of vaccination behavior, and the analyses reported above were designed to test that interpretation rather than merely assert it.

Three findings support the marker reading. First, the non-Islam group is extremely concentrated geographically: 83.2% of non-Islam-headed households are in Sumatera or Bali and Nusa Tenggara, and Java, which accounts for 45% of the sample, contributes only 36 such households. The comparison is therefore made largely between regions rather than within them, and the five broad island groupings available in IFLS-5 are too coarse to absorb the residual within-region clustering by province, ethnicity, and proximity to the private clinics through which the vaccine was exclusively sold. Second, and most tellingly, the association is not stable across regions: it is strong in Bali and Nusa Tenggara and Sulawesi, moderate in Sumatera, and reverses direction (with wide confidence intervals) in Java, with a significant test for effect modification (*p* = 0.002). A genuine individual-level effect of religious adherence on vaccination would not be expected to vary in direction across islands; a marker of local service environments and information ecologies would. Third, IFLS-5 records no measure of religiosity, religious practice, denomination within categories, or doctrinal position on vaccination, but only the stated affiliation of the household head, so the data cannot in principle identify a doctrinal mechanism.

One result deserves comment because it runs against the simplest version of the marker argument. The association strengthened rather than attenuated as region, place of residence, wealth, and maternal education were added (unadjusted OR 0.45; fully adjusted AOR 0.31). Confounding by the measured dimensions of socioeconomic position therefore does not explain the association, and we do not claim that it does; this contrasts with multi-country evidence from sub-Saharan Africa in which crude coverage differences by affiliation attenuate substantially after adjustment for wealth, education, and residence (36). What our data do show is that the association is geographically concentrated and regionally heterogeneous, which points to unmeasured local context, such as differences in vaccine promotion, provider recommendation, density of private providers stocking a self-paid product, and community information environments (14, 37), rather than to either measured socioeconomic position or religious belief. Concerns about vaccine ingredients and halal status have been documented in Indonesia and may contribute, but these are matters of product labeling, certification, and vaccine confidence that are amenable to policy, not fixed properties of belief.

Accordingly, we describe this finding as identifying population groups and places where uptake was lower and where tailored outreach is warranted, and we caution explicitly against reading it as evidence that Islamic belief or practice reduces vaccination. Framing it causally would risk stigmatizing the majority religious community in Indonesia while obscuring the modifiable service-delivery, financing, and communication barriers that most plausibly generate the observed pattern. The regional heterogeneity is itself the practical message: because the association is absent in Java and strong in eastern Indonesia, any response should be targeted to the specific provinces where the gap is real rather than applied to a religious group nationally.

Regional variation was also clear and persistent. Compared with Java, uptake was lower in Bali and Nusa Tenggara and higher in Sulawesi, and these associations remained stable in the sensitivity analyses. This suggests that rotavirus vaccine uptake was not evenly distributed across Indonesia. In a country with substantial geographic and social diversity, such variation is plausible and may reflect differences in service availability, provider distribution, local health promotion, transport barriers, and parental awareness of the vaccine (38, 39). The importance of region in this study indicates that national averages may conceal important local inequalities. It also suggests that strategies to improve uptake may need to be tailored to local contexts rather than applied uniformly across all regions.

Several additional associations present in the complete-case model proved not to be robust, and we now report them as such. Male sex, secondary maternal education, maternal age at delivery above 30 years, and paternal age 41–50 years all reached conventional significance in the fully adjusted complete-case model but lost it under multiple imputation, with point estimates moving toward the null. Because a reduced model containing only fully observed variables reproduces the same shift without any imputation, the divergence reflects systematic differences between the children retained in and excluded from the complete-case analysis rather than an artifact of the imputation procedure. These four associations should therefore be regarded as generated by, and confined to, the complete-case subsample. This is consistent with the cautions already warranted on other grounds: the confidence intervals for male sex (1.01–1.79) and secondary maternal education (0.49–0.97) were close to unity at one bound, maternal education showed no consistent gradient across categories, and male sex became significant only after later adjustment. We do not interpret these variables further, and note that the maternal education result in particular should not be read as evidence of an educational effect on rotavirus vaccine uptake in Indonesia (40, 41).

The correlates identified here are broadly consistent with evidence from other countries with a high burden of rotavirus diarrhea. In sub-Saharan Africa, social determinants such as caregiver education, household wealth, access to services, and exposure to health information have been linked to rotavirus vaccine uptake and to emerging vaccine “fatigue,” while expansion of rotavirus vaccination across the WHO African Region has been uneven across settings (32, 42, 43). The direction of the education association, however, is not uniform across settings: a facility-based study in southwest Nigeria found higher maternal education associated with greater rotavirus vaccine acceptability (44), whereas in the present study secondary maternal education was associated with lower uptake, underscoring that education may operate differently depending on local access, cost, and information environments. In Vietnam, behavioral and social drivers, including awareness and provider engagement, shaped uptake among a rural ethnic-minority population (45), and in Italy, determinants of uptake included parental education and regional differences in service delivery even within a high-income setting (46). Cost–benefit and impact analyses from sub-Saharan Africa and South America further show that rotavirus vaccination substantially reduces diarrheal disease burden and is generally cost-saving or cost-effective, reinforcing the value of improving uptake (42, 47). The convergence of these findings with our results, particularly the importance of prior health-service contact and contextual factors, suggests that strategies effective elsewhere may also be relevant to Indonesia. Taken together, evidence from within Southeast and East Asia—including the rural ethnic-minority population in Vietnam (45), self-paid uptake determinants in Japan (28), and post-introduction impact evaluation in Thailand (48)—provides a regional evidence base that is at least as relevant to the Indonesian context as the more extensively cited sub-Saharan African literature, and reinforces the recurring importance of health-service contact, information, and local context across diverse Asian settings.

Vaccination is only one component of rotavirus and diarrheal disease control. Adequate access to water, sanitation, and hygiene (WASH) facilities, together with promotion of handwashing, safe water, breastfeeding, and oral rehydration and zinc treatment, complements immunization by reducing transmission and the severity of diarrheal illness (49). Because rotavirus vaccine does not eliminate disease and uptake remained low during the study period, integrated infection prevention and control, combining vaccination with strengthened WASH infrastructure and hygiene promotion, is likely to be needed to achieve substantial reductions in childhood diarrhea in Indonesia.

These findings have practical and policy implications, which are now especially relevant given that rotavirus vaccine was introduced into Indonesia’s national immunization program in 2023 (8, 9, 12). First, the associations with complete basic immunization and postnatal care were the most robust in the study, surviving both multiple imputation and penalized estimation, and suggest that rotavirus vaccination should be delivered through, and actively linked to, existing maternal and child health services, especially routine immunization and postnatal care contacts (33, 50). Second, because religion of the household head marks local sociocultural and service context rather than a cause, and because the association is present in eastern Indonesia and Sumatera but absent in Java, the appropriate response is geographically targeted rather than directed at a religious group nationally: communication and demand-generation strategies should be locally adapted and co-developed with community and religious leaders in the specific provinces where gaps are observed, and should include clear information on vaccine safety, ingredients, and halal status where this is relevant to local concerns (6, 39, 51). Third, the regional variation implies that the national program should monitor coverage at subnational level and tailor implementation to local service realities rather than rely solely on a uniform approach. Fourth, sustaining and expanding coverage will require attention to supply, financing, and provider recommendation as the program scales up (52). Finally, policies to improve access and adherence to other infection prevention and control measures, particularly investment in WASH infrastructure and hygiene promotion, should be pursued alongside vaccination to maximize the impact on childhood diarrhea (49).

These findings also generate predictions that the national program can be used to test, which we state explicitly so that they can be checked rather than merely asserted. If the correlates identified here reflect the specific barriers of self-paid, private provision, then removing cost and dependence on private providers should attenuate them: the regional gradient and the differential associated with religion of the household head should narrow after national introduction, because both appear to index access to a privately sold product and the local information environment surrounding it. The association with prior immunization contact, by contrast, should persist, because it reflects engagement with health services rather than the ability to pay, and integration into routine immunization may even strengthen it. Should the regional and sociocultural differentials instead persist unchanged once the vaccine is free and programmatically delivered, that would indicate barriers which are not financial in origin and which demand a different policy response, directed at communication, trust, and provider practice rather than at availability. Post-introduction surveys with comparable measures would allow this pattern to be examined directly, and we suggest it as a concrete use of the baseline reported here.

It is also noteworthy that several variables often assumed to influence vaccination, such as wealth index, place of residence, maternal health insurance, and place of delivery, were not independently associated with uptake after adjustment. This does not mean that financial and access barriers are unimportant. Because the vaccine was self-paid during the survey period, affordability would be expected to matter; the absence of an association with wealth may instead reflect measurement limitations, residual confounding, or limited statistical power given the small number of vaccinated children, rather than a true absence of effect. More broadly, for a newer, self-paid vaccine outside the routine program, uptake may depend less on broad socioeconomic position alone and more on a combination of service contact, provider recommendation, information, and local availability (28, 53, 54).

The two variables excluded from the primary models because of sparse-data bias warrant explicit discussion, since their omission raises the possibility of residual confounding. Within the complete-case sample the problem was quasi-complete separation rather than mere sparseness: no child without antenatal care and no child with unmarried parents had received rotavirus vaccine, so maximum-likelihood estimation of their coefficients was not merely imprecise but divergent. Excluding them was therefore the correct immediate response, but it left the question of confounding unanswered. Penalized estimation allowed us to answer it: under Firth penalized likelihood both variables could be restored to the fully adjusted model, both proved null, and no principal estimate moved by more than 3.4% (Supplementary Table S9). Residual confounding by these two variables is therefore unlikely to be material. This is consistent with what their distributions would suggest: both are close to invariant in this population. Among children with the variable recorded, 98.9% of mothers had received antenatal care and 97.3% of children had married parents (90.7 and 95.0%, respectively, of the full analytic sample, the remainder being missing), leaving very little contrast available to confound, and with the fact that the pathway through which antenatal care would plausibly act is already represented by postnatal care, place of delivery, and maternal tetanus immunization, all of which were retained. We note nonetheless that the confidence intervals for both penalized estimates are extremely wide, so these analyses exclude only gross confounding, not modest confounding.

Sparse-data concerns extend to categories retained in the models. Maternal smoking (62 ever-smokers, 1.9%), paternal age above 50 years (63 children, 1.9%), and absence of maternal media access (205 children, 6.2%) are all thinly populated, and their wide confidence intervals are the visible signature of sparse data. Under penalization these estimates shrank toward the null, as expected, and none was significant; their point estimates should not be interpreted substantively. The principal findings are not affected, resting as they do on well-populated categories: 2,539 children had complete basic immunization, 1,252 had mothers who received postnatal care, 2,664 lived in Islam-headed households, and every region category contained at least 138 children. One caveat should be recorded, however: although the religion coefficient is stable under both imputation and penalization, it is identified by only 301 non-Islam-headed households and 46 vaccinated children within them, so it is more thinly supported than its narrow confidence interval alone conveys. That is a further reason to treat it as a contextual marker requiring corroboration rather than as a precise estimate.

This study has several strengths. It used IFLS, a large population-based dataset with broad national coverage, and included a wide range of child, maternal, household, contextual, and paternal variables. The hierarchical modeling strategy allowed associations to be examined sequentially, making it possible to distinguish findings that remained stable from those that emerged only after fuller adjustment. Most importantly, the principal findings were subjected to three independent robustness checks (multiple imputation across the full sample, penalized estimation restoring the separation-affected variables, and individual re-entry of the high-missingness variables) and survived all three, while four weaker associations did not and are reported as non-robust.

Several limitations should be acknowledged. First, vaccination status was based on caregiver report and was not verified against vaccination cards; recall error and misclassification are therefore possible, and the survey did not capture the number of doses, so the outcome reflects partial uptake rather than completion of the schedule. Second, the data were observational and cross-sectional, so the findings indicate associations rather than causal relationships, and the temporal ordering of correlates such as postnatal care and basic immunization relative to rotavirus vaccination cannot be confirmed. Third, IFLS-5 was conducted in 2014–2015, before rotavirus vaccine was introduced into the national program in 2023; the findings therefore describe a pre-introduction, self-paid context and may not reflect the current policy environment. Fourth, the analysis adopted a model-based (unweighted) approach rather than applying IFLS sampling weights, because the objective was to identify correlates of uptake rather than to generate nationally representative prevalence estimates, and the main design dimensions (region, urban or rural residence, and household wealth) were included as covariates; reassuringly, refitting the fully adjusted model with standard errors clustered on household left the estimates essentially unchanged.

Fifth, the primary models used a complete-case approach retaining 80.9% of the analytic sample. The identifying condition, that complete-case status is independent of the outcome given the covariates, was supported but not established: the outcome did not significantly predict complete-case status after adjustment, although the point estimate lay below unity. We therefore did not rest on that test alone: inverse-probability weighting reproduced the principal estimates almost exactly, and a tipping-point analysis showed that outcome-dependent selection would have to be considerably stronger than anything observed, and in fact stronger than any value we imposed, before the principal findings changed. Multiple imputation across all 3,320 children confirmed those findings while overturning four weaker ones, which we report as non-robust rather than as results. We note the limit of this reassurance: none of these analyses can address selection on variables that were never measured. Sixth, two variables (antenatal care and parental marital status) exhibited quasi-complete separation and could not be estimated by maximum likelihood. Penalized estimation restored them and found both null with no material change to the principal estimates, but their confidence intervals are very wide, so modest residual confounding cannot be excluded. Several retained categories are likewise sparse and their estimates are not interpreted. Seventh, the number of vaccinated children was relatively small (*n* = 287), which limited statistical power and warrants caution regarding overfitting; the fully adjusted model nonetheless showed adequate calibration (Hosmer-Lemeshow *p* = 0.626) and acceptable discrimination (AUC = 0.70), with modest pseudo-*R*^2^ values (Nagelkerke *R*^2^ = 0.095) as expected for socio-behavioral correlates of a low-prevalence outcome.

Eighth, although the broader childhood vaccination module spans children up to 14 years, rotavirus vaccination information in IFLS-5 was available only for children aged 1–5 years. Because age at interview and birth cohort are equivalent in a single-wave survey, the age term also serves as a birth-cohort term; neither specification was associated with uptake, and neither altered the other estimates. A single-wave design cannot, however, separate age, cohort, and period effects, so any secular change in private-market availability or awareness over 2009–2015 is absorbed into this single term, and the borderline likelihood-ratio test for the cohort specification (*p* = 0.053) cannot be resolved with these data. Ninth, the religion finding, although robust to imputation and penalization, is identified by a small and geographically concentrated group, varies significantly across regions, and is accompanied by no measure of religiosity or practice; it should be treated as a signal about places and contexts requiring investigation rather than as an estimate of a stable effect. Finally, several potentially important determinants of uptake, including parental knowledge of rotavirus, provider recommendation, vaccine confidence, concerns about vaccine ingredients, and direct measures of affordability or willingness to pay, were not available in the dataset.

Future research should move beyond identifying correlates and examine the mechanisms underlying them. In particular, the strong associations with religion of household head and region warrant more detailed investigation. Qualitative or mixed-methods studies could help clarify whether these patterns reflect differences in beliefs, trust, provider recommendation, service availability, affordability, or the way vaccine information is communicated. Further research should also examine how uptake changes as policy and financing environments shift, especially now that rotavirus vaccination has been integrated into the national immunization program.

## Conclusion

Rotavirus vaccine uptake among young Indonesian children was low during the IFLS-5 period. Four correlates proved robust across complete-case analysis, multiple imputation, and penalized estimation: complete basic immunization, postnatal care, religion of the household head, and geographic region. The latter two are best understood as markers of sociocultural, geographic, and health-service context rather than as causes of vaccination behavior, and the religion association was significantly heterogeneous across regions. Four weaker associations reported in the complete-case model did not survive multiple imputation and should not be relied upon. These findings suggest that improving rotavirus vaccine coverage in Indonesia will require more than vaccine availability alone: strategies are likely to be more effective if integrated into existing maternal and child health services, targeted to the specific regions where gaps are observed, and designed to address barriers related to awareness, acceptability, and access. Because the findings are based on cross-sectional, caregiver-reported data from before national introduction, they should be read as associations rather than causal effects, and as a baseline to be revisited as the national program matures.

## Data Availability

Publicly available datasets were analyzed in this study. This data can be found here: https://www.rand.org/health/surveys/FLS/IFLS.html.
