# Supplementary material for: Correlates of reported rotavirus vaccination among young Indonesian children: a cross-sectional analysis of the Indonesia Family Life Survey (IFLS-5)
Source: Front Public Health. 2026 Sep 18;14:1839949. doi: 10.3389/fpubh.2026.1839949 (PMC13631076; doi:10.3389/fpubh.2026.1839949)
Supplement: Supplementary file 1 [file Supplementary_file_1.docx]

**SUPPLEMENTARY FILES**

**Supplementary Table S1. Complete list of the 28 candidate variables considered for the hierarchical logistic regression models, grouped by conceptual domain.**

| **Domain** | **Variable** | **Status in primary hierarchical models** | **Reason if excluded** |
| --- | --- | --- | --- |
| Child-related | Sex of child | Included (Model 1) |  |
|  | Birth order | Included (Model 1) |  |
|  | Basic immunization status | Included (Model 1) |  |
|  | Child health insurance | Excluded | Missing data >10% (74.4%) |
| Maternal/perinatal healthcare | Antenatal care | Excluded | Quasi-complete separation / sparse-data bias; restored under Firth penalized likelihood (Supplementary Table S9) |
|  | Postnatal care | Included (Model 2) |  |
|  | Place of delivery | Included (Model 2) |  |
|  | Maternal tetanus immunization during pregnancy | Included (Model 2) |  |
| Maternal sociodemographic | Maternal age at delivery | Included (Model 3) |  |
|  | Maternal age at survey | Excluded | Conceptual overlap with maternal age at delivery |
|  | Parental marital status | Excluded | Quasi-complete separation / sparse-data bias; restored under Firth penalized likelihood (Supplementary Table S9) |
|  | Maternal education | Included (Model 3) |  |
|  | Maternal employment | Included (Model 3) |  |
|  | Maternal health insurance | Included (Model 3) |  |
|  | Maternal smoking status | Included (Model 3) |  |
|  | Maternal media access | Included (Model 3) |  |
| Household/contextual | Religion of household head | Included (Model 4) |  |
|  | Household size | Included (Model 4) |  |
|  | Wealth index | Included (Model 4) |  |
|  | Place of residence | Included (Model 4) |  |
|  | Region | Included (Model 4) |  |
|  | Decision-maker for child healthcare | Excluded | Missing data >10% (26.3%) |
| Paternal | Paternal age at survey | Included (Model 5) |  |
|  | Paternal education | Excluded | Missing data >10% (10.7%) |
|  | Paternal employment | Excluded | Missing data >10% (10.2%) |
|  | Paternal health insurance | Excluded | Missing data >10% (25.9%) |
|  | Paternal smoking status | Excluded | Missing data >10% (25.9%) |
|  | Paternal media access | Included (Model 5) |  |

Note: Child age was included as a continuous covariate in all hierarchical models and is presented separately in Table 1; it is not counted among the 28 categorical/binary candidate variables above. Of the 28 candidate variables, 19 were retained in the primary hierarchical models (Models 1–5); the remaining 9 were excluded for the reasons indicated.

**Supplementary Table S2. Comparison of children included in versus excluded from the fully adjusted model (complete-case analysis).**

| **Characteristic** | **Included (*n* = 2,686)** | **Excluded (*n* = 634)** | ***p*-value** |
| --- | --- | --- | --- |
| **Rotavirus vaccination, ≥1 dose, *n* (%)** | 222 (8.3) | 65 (10.3) | 0.128 |
| **Child age, years, mean (SD)** | 3.16 (1.38) | 3.34 (1.38) | 0.003 |
| **Sex of child, *n* (%)** |  |  | 0.844 |
| Female | 1,316 (49.0) | 314 (49.5) |  |
| Male | 1,370 (51.0) | 320 (50.5) |  |
| **Religion of household head, *n* (%)** |  |  | 0.185 |
| Non-Islam | 301 (11.2) | 26 (8.5) |  |
| Islam | 2,385 (88.8) | 279 (91.5) |  |
| **Basic immunization status, *n* (%)** |  |  | 0.015 |
| Incomplete | 608 (22.6) | 173 (27.3) |  |
| Complete | 2,078 (77.4) | 461 (72.7) |  |
| **Region, *n* (%)** |  |  | <0.001 |
| Java | 1,136 (42.3) | 376 (59.3) |  |
| Sumatera | 888 (33.1) | 124 (19.6) |  |
| Bali and Nusa Tenggara | 416 (15.5) | 90 (14.2) |  |
| Kalimantan | 125 (4.7) | 13 (2.1) |  |
| Sulawesi | 121 (4.5) | 31 (4.9) |  |
| **Place of residence, *n* (%)** |  |  | 0.112 |
| Rural | 1,142 (42.5) | 247 (39.0) |  |
| Urban | 1,544 (57.5) | 387 (61.0) |  |
| **Wealth index, *n* (%)** |  |  | 0.266 |
| Poorest | 488 (18.2) | 114 (18.1) |  |
| Poorer | 457 (17.0) | 100 (15.9) |  |
| Middle | 616 (22.9) | 163 (25.9) |  |
| Richer | 536 (20.0) | 106 (16.9) |  |
| Richest | 589 (21.9) | 146 (23.2) |  |

Note: Included children had complete data on all variables in the fully adjusted model (*n* = 2,686); excluded children had missing data on at least one such variable (*n* = 634). Percentages are column percentages calculated among non-missing observations within each group; for religion of household head, valid data were available for all included children and for 305 excluded children. *p*-values are from the chi-square test for categorical variables and the independent-samples t-test for child age. Reported uptake did not differ significantly between groups; the principal differences were geographic (excluded children more often from Java), with a smaller difference in completed basic immunization, indicating that the complete-case approach is unlikely to have introduced major selection bias.

**Supplementary Table S3. Generalized variance inflation factors (GVIF) for the 19 predictors in the fully adjusted model.**

| **Variable** | **Degrees of freedom (Df)** | **GVIF** | **Adjusted GVIF [GVIF^(1/(2·Df))]²** |
| --- | --- | --- | --- |
| Sex of child | 1 | 1.01 | 1.01 |
| Birth order | 1 | 1.18 | 1.18 |
| Basic immunization status | 1 | 1.05 | 1.05 |
| Postnatal care | 1 | 1.06 | 1.06 |
| Place of delivery | 2 | 1.37 | 1.17 |
| Maternal tetanus immunization during pregnancy | 1 | 1.05 | 1.05 |
| Maternal age at delivery | 2 | 1.78 | 1.34 |
| Maternal education | 2 | 1.37 | 1.17 |
| Maternal employment | 1 | 1.09 | 1.09 |
| Maternal health insurance | 1 | 1.05 | 1.05 |
| Maternal smoking status | 1 | 1.05 | 1.05 |
| Maternal media access | 1 | 1.48 | 1.48 |
| Religion of household head | 1 | 1.16 | 1.16 |
| Household size | 2 | 1.25 | 1.12 |
| Wealth index | 4 | 1.23 | 1.05 |
| Place of residence | 1 | 1.25 | 1.25 |
| Region | 4 | 1.46 | 1.10 |
| Paternal age at survey | 3 | 1.72 | 1.20 |
| Paternal media access | 1 | 1.44 | 1.44 |

Note: Generalized variance inflation factors (GVIF) were used because several predictors are categorical with more than one degree of freedom (Df); for such predictors, conventional VIF calculated on individual dummy-coded contrasts can be misleading, so the adjusted GVIF, [GVIF^(1/(2·Df))]², is reported for comparability with the conventional VIF scale, following the approach of Fox and Monette (1992). Across all 19 predictors, the mean adjusted GVIF was 1.16 and the maximum was 1.48 (maternal media access), both well below the conventional threshold of concern (5 or 10), indicating no meaningful multicollinearity in the fully adjusted model. Fox J, Monette G. Generalized collinearity diagnostics. J Am Stat Assoc. (1992) 87:178–83. doi: 10.1080/01621459.1992.10475190.

**Supplementary Table S4. Fully adjusted model (Model 5) with standard versus household-clustered standard errors: adjusted odds ratios and 95% confidence intervals.**

| **Characteristic** | **AOR** | **95% CI (standard)** | **95% CI (clustered by household)** |
| --- | --- | --- | --- |
| **Child age, per year (continuous)** | 1.02 | 0.91–1.13 | 0.91–1.13 |
| **Sex of child (Ref Female)** |  |  |  |
| Male | 1.34 | 1.01–1.79* | 1.01–1.79* |
| **Birth order (Ref First born)** |  |  |  |
| Second born and later | 1.03 | 0.75–1.42 | 0.74–1.42 |
| **Basic immunization status (Ref Incomplete)** |  |  |  |
| Complete | 2.71 | 1.73–4.24* | 1.72–4.27* |
| **Postnatal care (Ref No)** |  |  |  |
| Yes | 1.57 | 1.17–2.11* | 1.15–2.15* |
| **Place of delivery (Ref Home)** |  |  |  |
| Health facility | 1.26 | 0.83–1.91 | 0.81–1.96 |
| Other | 1.33 | 0.88–2.03 | 0.86–2.07 |
| **Maternal tetanus immunization during pregnancy (Ref No)** |  |  |  |
| Yes | 1.28 | 0.89–1.82 | 0.87–1.86 |
| **Maternal age at delivery (Ref ≤20 years)** |  |  |  |
| 21–30 years | 0.93 | 0.56–1.53 | 0.56–1.54 |
| >30 years | 1.85 | 1.02–3.37* | 0.99–3.44 |
| **Maternal education (Ref Primary)** |  |  |  |
| Secondary | 0.69 | 0.49–0.97* | 0.48–0.98* |
| Tertiary | 0.67 | 0.42–1.06 | 0.40–1.10 |
| **Maternal employment (Ref Unemployed)** |  |  |  |
| Employed | 1.02 | 0.76–1.39 | 0.74–1.42 |
| **Maternal health insurance (Ref No)** |  |  |  |
| Yes | 1.01 | 0.75–1.35 | 0.74–1.37 |
| **Maternal smoking status (Ref Never)** |  |  |  |
| Ever | 1.70 | 0.67–4.30 | 0.68–4.26 |
| **Maternal media access (Ref No)** |  |  |  |
| Yes | 0.95 | 0.47–1.93 | 0.50–1.80 |
| **Religion of household head (Ref Non-Islam)** |  |  |  |
| Islam | 0.31 | 0.21–0.47* | 0.20–0.48* |
| **Household size (Ref ≤4 persons)** |  |  |  |
| 5–9 persons | 0.81 | 0.57–1.14 | 0.56–1.16 |
| ≥10 persons | 1.22 | 0.76–1.98 | 0.74–2.02 |
| **Wealth index (Ref Poorest)** |  |  |  |
| Poorer | 1.21 | 0.73–2.01 | 0.72–2.04 |
| Middle | 1.15 | 0.72–1.86 | 0.70–1.87 |
| Richer | 1.38 | 0.85–2.26 | 0.82–2.33 |
| Richest | 1.39 | 0.86–2.25 | 0.84–2.29 |
| **Place of residence (Ref Rural)** |  |  |  |
| Urban | 0.94 | 0.68–1.29 | 0.68–1.30 |
| **Region (Ref Java)** |  |  |  |
| Sumatera | 0.79 | 0.54–1.15 | 0.52–1.19 |
| Bali and Nusa Tenggara | 0.51 | 0.31–0.84* | 0.31–0.84* |
| Kalimantan | 1.33 | 0.69–2.56 | 0.68–2.59 |
| Sulawesi | 2.81 | 1.64–4.81* | 1.61–4.89* |
| **Paternal age at survey (Ref ≤30 years)** |  |  |  |
| 31–40 years | 0.76 | 0.52–1.11 | 0.51–1.12 |
| 41–50 years | 0.45 | 0.25–0.81* | 0.25–0.83* |
| >50 years | 0.45 | 0.14–1.45 | 0.14–1.41 |
| **Paternal media access (Ref No)** |  |  |  |
| Yes | 1.07 | 0.45–2.58 | 0.46–2.49 |

Note: The model was fitted to 2,686 children in 2,462 households. AORs are identical under both approaches; only the standard errors, and therefore the confidence intervals, differ. Cluster-robust standard errors account for non-independence among children from the same household. The confidence intervals are materially unchanged across the two approaches, indicating that the inferences are not sensitive to within-household clustering. *Significant (*p* < 0.05).

**Supplementary Table S5. Birth-cohort (single-year age) specification of the fully adjusted model (Model 5): adjusted odds ratios for rotavirus vaccine uptake.**

| **Birth cohort (age at interview)** | **n** | **Uptake, *n* (%)** | **AOR (95% CI)** | ***p*-value** |
| --- | --- | --- | --- | --- |
| 2013–14 (1 year) | 433 | 27 (6.2) | 1.00 (reference) | - |
| 2012–13 (2 years) | 485 | 52 (10.7) | 1.95 (1.18–3.22) | 0.009 |
| 2011–12 (3 years) | 574 | 51 (8.9) | 1.64 (0.99–2.73) | 0.055 |
| 2010–11 (4 years) | 611 | 50 (8.2) | 1.48 (0.88–2.47) | 0.138 |
| 2009–10 (5 years) | 583 | 42 (7.2) | 1.38 (0.80–2.36) | 0.247 |

Note: Fitted to the 2,686 children in the fully adjusted model, adjusted for all Model 5 covariates. In a single-wave survey age at interview and birth cohort are equivalent, so single-year age indicators are birth-cohort indicators; birth-year labels are approximate because IFLS-5 fieldwork ran from late 2014 into 2015. Likelihood-ratio test comparing this categorical specification with the continuous specification: χ² = 7.67, df = 3, *p* = 0.053. No adjusted odds ratio elsewhere in Model 5 changed by more than 0.04 under this specification. Uptake percentages shown are for these 2,686 children; the unadjusted comparison of uptake across single years of age reported in the Results (χ² = 4.89, df = 4, *p* = 0.30) is calculated on all 3,320 children in the analytic sample.

**Supplementary Table S6. Religion of the household head as a contextual marker: geographic distribution, region-stratified associations, and sequential adjustment.**

**Panel A. Distribution of non-Islam-headed households and uptake, by region**

| **Region** | **Non-Islam households, n (% of region)** | **% of all non-Islam households** | **Uptake % non-Islam** | **Uptake % Islam** | **Crude OR Islam vs non-Islam (95% CI)** |
| --- | --- | --- | --- | --- | --- |
| Java | 36 (2.7) | 11.0 | 2.8 | 9.0 | 3.46 (0.47–25.52) |
| Sumatera | 146 (15.2) | 44.6 | 13.0 | 6.2 | 0.44 (0.25–0.77) |
| Bali and Nusa Tenggara | 126 (28.6) | 38.5 | 16.7 | 2.9 | 0.15 (0.07–0.33) |
| Kalimantan | 1 (0.8) | 0.3 | 100.0 | 8.5 | Not estimable |
| Sulawesi | 18 (12.9) | 5.5 | 50.0 | 14.9 | 0.17 (0.06–0.50) |

**Panel B. Religion coefficient under sequential adjustment**

| **Model** | **n** | **AOR for Islam-headed household (95% CI)** |
| --- | --- | --- |
| Unadjusted (religion only) | 2,991 | 0.45 (0.32–0.63) |
| + region | 2,991 | 0.36 (0.25–0.51) |
| + region, place of residence | 2,991 | 0.36 (0.25–0.52) |
| + region, residence, wealth index | 2,987 | 0.35 (0.25–0.51) |
| + region, residence, wealth, maternal education | 2,925 | 0.34 (0.24–0.50) |
| Fully adjusted (Model 5) | 2,686 | 0.31 (0.21–0.47) |

Note: Panel A is restricted to the 2,991 children with religion of household head recorded. Kalimantan contains a single non-Islam-headed household, so its stratum-specific odds ratio is not estimable. Test for effect modification of religion by region (Firth penalized likelihood-ratio test, Kalimantan excluded): χ² = 14.55, df = 3, *p* = 0.002. In the complete-case analytic sample the religion contrast is identified by 301 non-Islam-headed households containing 46 vaccinated children. Panel B shows that the association strengthens rather than attenuates with adjustment, indicating that it is not explained by the measured dimensions of socioeconomic position.

**Supplementary Table S7. Robustness of the complete-case estimates to selection on observed variables: inverse-probability weighting and tipping-point analysis.**

**Panel A. Fully adjusted model, unweighted versus inverse-probability weighted**

| **Characteristic** | **Complete case, AOR (95% CI)** | **Inverse-probability weighted, AOR (95% CI)** |
| --- | --- | --- |
| Complete basic immunization (ref Incomplete) | 2.71 (1.73–4.24) | 2.60 (1.76–3.83) |
| Postnatal care: Yes (ref No) | 1.57 (1.17–2.11) | 1.56 (1.20–2.02) |
| Religion of household head: Islam (ref Non-Islam) | 0.31 (0.21–0.47) | 0.32 (0.23–0.46) |
| Region: Bali and Nusa Tenggara (ref Java) | 0.51 (0.31–0.84) | 0.51 (0.34–0.78) |
| Region: Sulawesi (ref Java) | 2.81 (1.64–4.81) | 2.89 (1.83–4.59) |
| Male (ref Female) | 1.34 (1.01–1.79) | 1.36 (1.05–1.75) |
| Maternal education: secondary (ref Primary) | 0.69 (0.49–0.97) | 0.68 (0.50–0.92) |
| Maternal age at delivery >30 (ref ≤20) | 1.85 (1.02–3.37) | 1.90 (1.13–3.21) |
| Paternal age 41–50 (ref ≤30) | 0.45 (0.25–0.81) | 0.47 (0.29–0.77) |

**Panel B. Tipping-point analysis: principal associations under imposed outcome-dependent missingness**

| **Assumed OR, outcome predicting completeness** | **Basic immunization** | **Postnatal care** | **Islam-headed household** | **Bali and Nusa Tenggara** | **Sulawesi** |
| --- | --- | --- | --- | --- | --- |
| 0.40 | 2.46 (1.74–3.48) | 1.55 (1.22–1.96) | 0.33 (0.24–0.45) | 0.49 (0.33–0.72) | 2.81 (1.85–4.29) |
| 0.55 | 2.53 (1.75–3.66) | 1.55 (1.21–1.99) | 0.33 (0.23–0.45) | 0.50 (0.33–0.75) | 2.86 (1.84–4.44) |
| 0.70 | 2.58 (1.76–3.78) | 1.55 (1.20–2.01) | 0.32 (0.23–0.45) | 0.51 (0.34–0.77) | 2.89 (1.84–4.54) |
| 0.77 | 2.60 (1.76–3.82) | 1.56 (1.20–2.02) | 0.32 (0.23–0.45) | 0.51 (0.34–0.78) | 2.90 (1.84–4.58) |
| 1.00 | 2.64 (1.77–3.94) | 1.56 (1.19–2.03) | 0.32 (0.22–0.46) | 0.52 (0.34–0.80) | 2.93 (1.83–4.68) |
| 1.50 | 2.70 (1.78–4.09) | 1.56 (1.19–2.05) | 0.32 (0.22–0.46) | 0.53 (0.34–0.83) | 2.96 (1.83–4.80) |

Note: Panel A weights each complete case by the reciprocal of its estimated probability of being a complete case given the fully observed covariates and the outcome (weight range 1.06 to 2.21, mean 1.24); robust (HC1) standard errors. Panel B imposes outcome-dependent missingness of the stated magnitude, reweights the complete cases accordingly and refits. The observed value from the direct test was 0.77 (95% CI 0.57–1.04), so 0.40 represents selection considerably stronger than anything supported by the data. All five principal associations retain direction, magnitude and significance throughout. These analyses assess selection on observed variables only; because they reweight the complete cases they cannot recover information carried by the 634 excluded children, which is the separate question addressed by multiple imputation (Supplementary Table S8). Panels A and B are separate model fits, so the row at the observed value of 0.77 in Panel B is not expected to reproduce the inverse-probability-weighted estimates of Panel A exactly.

**Supplementary Table S8. Fully adjusted model (Model 5): complete-case analysis (*n* = 2,686) compared with multiple imputation by chained equations (*n* = 3,320, *m* = 20).**

| **Characteristic** | **Complete case, AOR (95% CI)** | **Multiple imputation, AOR (95% CI)** | ***p*-value (MI)** |
| --- | --- | --- | --- |
| Male (ref Female) | 1.34 (1.01–1.79) | 1.13 (0.88–1.45) | 0.337 |
| Second born or later (ref First born) | 1.03 (0.75–1.42) | 0.93 (0.69–1.25) | 0.642 |
| Complete basic immunization (ref Incomplete) | 2.71 (1.73–4.24) | 2.78 (1.88–4.11) | <0.001 |
| Postnatal care: Yes (ref No) | 1.57 (1.17–2.11) | 1.40 (1.06–1.85) | 0.016 |
| Delivery: health facility (ref Home) | 1.26 (0.83–1.91) | 1.31 (0.89–1.92) | 0.171 |
| Delivery: other (ref Home) | 1.33 (0.88–2.03) | 1.32 (0.90–1.95) | 0.156 |
| Tetanus immunization in pregnancy: Yes (ref No) | 1.28 (0.89–1.82) | 1.17 (0.84–1.62) | 0.360 |
| Maternal age at delivery 21–30 (ref ≤20) | 0.93 (0.56–1.53) | 0.89 (0.57–1.39) | 0.608 |
| Maternal age at delivery >30 (ref ≤20) | 1.85 (1.02–3.37) | 1.56 (0.90–2.70) | 0.112 |
| Maternal education: secondary (ref Primary) | 0.69 (0.49–0.97) | 0.82 (0.61–1.11) | 0.204 |
| Maternal education: tertiary (ref Primary) | 0.67 (0.42–1.06) | 0.78 (0.52–1.17) | 0.239 |
| Maternal employment: employed (ref Unemployed) | 1.02 (0.76–1.39) | 0.98 (0.75–1.28) | 0.877 |
| Maternal health insurance: yes (ref No) | 1.01 (0.75–1.35) | 1.00 (0.76–1.31) | 0.998 |
| Maternal smoking: ever (ref Never) | 1.70 (0.67–4.30) | 1.40 (0.62–3.17) | 0.422 |
| Maternal media access: yes (ref No) | 0.95 (0.47–1.93) | 0.90 (0.47–1.72) | 0.760 |
| Religion of household head: Islam (ref Non-Islam) | 0.31 (0.21–0.47) | 0.32 (0.22–0.48) | <0.001 |
| Household size 5–9 (ref ≤4) | 0.81 (0.57–1.14) | 0.89 (0.66–1.20) | 0.457 |
| Household size ≥10 (ref ≤4) | 1.22 (0.76–1.98) | 1.29 (0.87–1.92) | 0.206 |
| Wealth: poorer (ref Poorest) | 1.21 (0.73–2.01) | 1.05 (0.68–1.63) | 0.815 |
| Wealth: middle (ref Poorest) | 1.15 (0.72–1.86) | 0.96 (0.63–1.45) | 0.845 |
| Wealth: richer (ref Poorest) | 1.38 (0.85–2.26) | 1.20 (0.79–1.84) | 0.393 |
| Wealth: richest (ref Poorest) | 1.39 (0.86–2.25) | 1.18 (0.78–1.79) | 0.438 |
| Urban residence (ref Rural) | 0.94 (0.68–1.29) | 0.99 (0.75–1.31) | 0.938 |
| Region: Sumatera (ref Java) | 0.79 (0.54–1.15) | 0.86 (0.62–1.19) | 0.366 |
| Region: Bali and Nusa Tenggara (ref Java) | 0.51 (0.31–0.84) | 0.61 (0.39–0.93) | 0.023 |
| Region: Kalimantan (ref Java) | 1.33 (0.69–2.56) | 1.25 (0.66–2.39) | 0.489 |
| Region: Sulawesi (ref Java) | 2.81 (1.64–4.81) | 2.44 (1.51–3.96) | <0.001 |
| Paternal age 31–40 (ref ≤30) | 0.76 (0.52–1.11) | 0.88 (0.61–1.27) | 0.501 |
| Paternal age 41–50 (ref ≤30) | 0.45 (0.25–0.81) | 0.60 (0.34–1.04) | 0.068 |
| Paternal age >50 (ref ≤30) | 0.45 (0.14–1.45) | 0.88 (0.35–2.19) | 0.780 |
| Paternal media access: yes (ref No) | 1.07 (0.45–2.58) | 1.52 (0.67–3.46) | 0.322 |
| Child age, per year | 1.02 (0.91–1.13) | 1.01 (0.92–1.11) | 0.827 |

Note: AOR = adjusted odds ratio. Multiple imputation used fully conditional specification with 10 cycles, logistic and multinomial logistic imputation models with a weak ridge penalty, the outcome included as a predictor in every imputation model, and bootstrap-based parameter draws; results were pooled using Rubin’s rules with Barnard-Rubin adjusted degrees of freedom. The median fraction of missing information was 0.02. Four associations significant in the complete-case model lost significance under imputation: male sex, secondary maternal education, maternal age at delivery above 30 years, and paternal age 41–50 years. A reduced model containing only fully observed variables reproduces the same shift with no imputation (male sex OR 1.14 in all 3,320 children versus 1.37 in the 2,686 complete cases), indicating a selection effect rather than an imputation artefact.

**Supplementary Table S9. Firth penalized logistic regression of the fully adjusted model with antenatal care and parental marital status restored (*n* = 2,686; 222 vaccinated children).**

| **Characteristic** | **Maximum likelihood, AOR (95% CI)** | **Firth penalized, AOR (95% CI)** | ***p*-value** |
| --- | --- | --- | --- |
| Antenatal care: yes (ref No) | Not estimable* | 1.69 (0.18–10.07) | 0.710 |
| Parents married (ref Unmarried/separated/widowed) | Not estimable* | 2.10 (0.26–9.95) | 0.571 |
| Complete basic immunization (ref Incomplete) | 2.71 (1.73–4.24) | 2.62 (1.72–4.16) | <0.001 |
| Postnatal care: Yes (ref No) | 1.57 (1.17–2.11) | 1.55 (1.16–2.08) | 0.003 |
| Religion of household head: Islam (ref Non-Islam) | 0.31 (0.21–0.47) | 0.32 (0.21–0.48) | <0.001 |
| Region: Bali and Nusa Tenggara (ref Java) | 0.51 (0.31–0.84) | 0.52 (0.31–0.84) | 0.007 |
| Region: Sulawesi (ref Java) | 2.81 (1.64–4.81) | 2.76 (1.60–4.64) | <0.001 |
| Maternal education: secondary (ref Primary) | 0.69 (0.49–0.97) | 0.69 (0.49–0.96) | 0.029 |
| Male (ref Female) | 1.34 (1.01–1.79) | 1.33 (1.00–1.77) | 0.047 |
| Maternal age at delivery >30 (ref ≤20) | 1.85 (1.02–3.37) | 1.82 (1.01–3.33) | 0.044 |
| Paternal age 41–50 (ref ≤30) | 0.45 (0.25–0.81) | 0.45 (0.25–0.80) | 0.006 |
| Paternal age >50 (ref ≤30) | 0.45 (0.14–1.45) | 0.49 (0.14–1.37) | 0.183 |
| Maternal smoking: ever (ref Never) | 1.70 (0.67–4.30) | 1.76 (0.66–4.03) | 0.240 |
| Maternal media access: yes (ref No) | 0.95 (0.47–1.93) | 0.92 (0.47–1.90) | 0.806 |
| Child age, per year | 1.02 (0.91–1.13) | 1.02 (0.91–1.13) | 0.780 |

Note: *Antenatal care and parental marital status could not be estimated by maximum likelihood because of quasi-complete separation: none of the 9 children without antenatal care and none of the 10 children with unmarried parents in the complete-case sample had received rotavirus vaccine (maximum-likelihood coefficients diverged to 28.7 and 26.0 with standard errors of 1.96 × 10⁶ and 4.56 × 10⁵, respectively). Firth estimation applies a Jeffreys-prior penalty that removes the first-order bias of maximum likelihood and yields finite estimates under separation. Confidence intervals are profile penalized-likelihood intervals and *p*-values are from penalized likelihood-ratio tests. Restoring the two separation-affected variables changed no principal estimate by more than 3.4%.
